# Transplantation of Nerve from Rabbit to Man

**Published:** 1888-06

**Authors:** 


### TRANSPLANTATION OF NERVE FROM THE RABBIT TO MAN.

Dr. Gersung, of Vienna, assistant to Professor Billroth, has recently performed a novel and interesting operation—the transplantation of nerve from the rabbit to man. The case has not hitherto been published in any medical journal, but owing to its general interest the bare fact had found its way into the lay newspapers. Our Vienna correspondent has received from Dr. Gersung a verbal account of the salient points of this most remarkable operation, which has so far been conspicuously successful. The patient is Professor von Fleischl, the distinguished occupant of the chair of physiology in the University of Vienna; sixteen years ago he accidentally wounded himself while couducting a *post-mortem* examination, and severe inflammation of the whole right upper limb ensued. During the course of the disease the terminal phalanx of the thumb became gangrenous. The stump thus left was painful, and later on re-amputation was performed. This was followed by the formation of neuromata. For this condition the branches of the median nerve which supply the thumb were first resected, together with the terminal neuromata, and at a later period, when new neuromata began to develop, the central parts of the same nerves, together with the branches of the radial nerve, which supply the thumb, were resected. Fresh neuromata now developed on the branches of the median nerve, which were treated, without any success whatever, by the injection of hyperosmic acid and electrolysis. Two years ago the neuromata were resected again, and the resection of the nerves was continued as far as the "ligamentum carpi volare"; on this occasion, the branches which supply the radial and the ulnar sides of the index, as well as the radial side of the middle finger, were resected to a great extent. The forefinger now became anæsthetic, except the dorsal aspect of its first phalanx, which, as is known, is supplied by the radial nerve; in the same way the whole radial side of the middle finger became anæsthetic. The pain, however, again recurred, as after the previous operations, and during the course of the second week after the last operation, the patient became aware that a fresh neuroma was developing. The suffering finally became so severe that the patient wished to undergo another operation, in order to procure, at least temporary relief. Accordingly, the following operation was performed: On March 4 the patient was put under the influence of chloroform, and the neuroma, which was situated behind the volar carpal ligament, was excised, the nerve being cut through behind the neuroma. The peripheral nerve stumps of the two digital branches above mentioned were then sought for. A rabbit was now killed, and as long a piece as possible of the sciatic nerve of the animal, with the two branches into which it becomes divided, was dissected from it (the animal still presenting voluntary contractions). The sciatic nerve was afterwards inserted into the space between the central stump of the median nerve and its digital branches; the central end of the sciatic nerve was sutured to the connective tissue which covered the median nerve, and the two branches were sutured to the digital branches of the median nerve; the portion of nerve, measuring about six centimètres, which was deficient, was thus made up. After the operation severe pain persisted for some hours, but then entirely subsided. Healing took place by first intention. As two months have now elapsed since the date of operation and the pain has not returned, it may be hoped that the favorable result will become a permanent one. Sensibility, moreover, is becoming re-established in the part. Dr. Gersung has postponed the publication of the case, because he wished to observe whether complete sensibility would return; he hopes with confidence that this will be the case. The ultimate result will be awaited with great interest; for if it is as favorable as now appears probable Dr. Gersung's recommendation that the operation should be given an extended trial will doubtless be widely acted on.—*British Medical Journal.*

